# Unravelling the neurophysiological basis of aggression in a fish model

**DOI:** 10.1186/1471-2164-11-498

**Published:** 2010-09-16

**Authors:** Amy L Filby, Gregory C Paull, Tamsin FA Hickmore, Charles R Tyler

**Affiliations:** 1 School of Biosciences, University of Exeter, Hatherly Laboratories, Prince of Wales Road, Exeter, Devon EX4 4PS, UK

## Abstract

**Background:**

Aggression is a near-universal behaviour with substantial influence on and implications for human and animal social systems. The neurophysiological basis of aggression is, however, poorly understood in all species and approaches adopted to study this complex behaviour have often been oversimplified. We applied targeted expression profiling on 40 genes, spanning eight neurological pathways and in four distinct regions of the brain, in combination with behavioural observations and pharmacological manipulations, to screen for regulatory pathways of aggression in the zebrafish (*Danio rerio*), an animal model in which social rank and aggressiveness tightly correlate.

**Results:**

Substantial differences occurred in gene expression profiles between dominant and subordinate males associated with phenotypic differences in aggressiveness and, for the chosen gene set, they occurred mainly in the hypothalamus and telencephalon. The patterns of differentially-expressed genes implied multifactorial control of aggression in zebrafish, including the hypothalamo-neurohypophysial-system, serotonin, somatostatin, dopamine, hypothalamo-pituitary-interrenal, hypothalamo-pituitary-gonadal and histamine pathways, and the latter is a novel finding outside mammals. Pharmacological manipulations of various nodes within the hypothalamo-neurohypophysial-system and serotonin pathways supported their functional involvement. We also observed differences in expression profiles in the brains of dominant versus subordinate females that suggested sex-conserved control of aggression. For example, in the HNS pathway, the gene encoding arginine vasotocin (AVT), previously believed specific to male behaviours, was amongst those genes most associated with aggression, and AVT inhibited dominant female aggression, as in males. However, sex-specific differences in the expression profiles also occurred, including differences in aggression-associated tryptophan hydroxylases and estrogen receptors.

**Conclusions:**

Thus, through an integrated approach, combining gene expression profiling, behavioural analyses, and pharmacological manipulations, we identified candidate genes and pathways that appear to play significant roles in regulating aggression in fish. Many of these are novel for non-mammalian systems. We further present a validated system for advancing our understanding of the mechanistic underpinnings of complex behaviours using a fish model.

## Background

The display of aggression is a near-universal trait in the animal kingdom, of which several forms have been distinguished [1]. In non-human animals, aggression typically occurs in the context of competition for limited resources, including food, mates and nesting sites, where it is important in the establishment of territories and dominance hierarchies. In this respect, aggression is viewed as an adaptation that can have key effects on the lifelong success of individuals and that conveys evolutionary fitness [2]. In humans, expression of aggression out of the appropriate context is, in contrast, currently recognised as one of the greatest and most challenging problems in society and has been identified as a key area for research and pharmacological intervention [3,4]. Despite the impacts of aggression in social systems and the plethora of studies in this area, its neurophysiological basis is not well understood in any species.

One factor likely contributing to the limited knowledge on the control of aggression is that studies in this area have generally focused on linking specific neurotransmitters/neuroendocrine factors from individual pathways with the expression of aggressive behaviours. This approach does not build an understanding of the combination of factors and their interactions that form the biological determinants of this behaviour. Given that aggression is a complex behavioural display, multifactorial control is expected [5]. Gene expression profiling, therefore, offers considerable potential for unravelling the complexities of its control. This is because the expression of many genes representing different pathways can be assessed simultaneously in the same individual [6,7]. Expression profiling approaches to studying the brain have already enhanced knowledge in other areas of behavioural science, including learning and memory, courtship behaviour and mate choice, reward behaviour, and disorders such as depression, schizophrenia and Parkinson's disease [8-12]. Only recently, however, has this approach begun to be applied in the study of aggression. Moreover, this work has largely focused on a few select laboratory models (*Drosophila *and mouse [13-18]), although has also included the chicken [19] and honey bee [7].

The zebrafish (*Danio rerio*) is used extensively for research on development and medicine [20], and is now also emerging as a model for studying animal behaviour [21]. The zebrafish is a group-living species in which dominance hierarchies form in both sexes (at least in captivity). Aggression is used commonly by dominant individuals to enable them to occupy territories over spawning sites and protect their status from their subordinates [22-24]. This close association between aggression and social rank, together with the zebrafish's less complex behavioural patterns compared with mammals, make the zebrafish highly appropriate for mechanistic studies for understanding aggression. Surprisingly, despite the wealth of studies quantifying aggression in zebrafish and the potential for the application of genomic tools for understanding the mechanisms of this behaviour, only one study (and investigating only a single neuropeptide) has explored its neurophysiological underpinnings [22].

In this study, we set out to investigate the neurophysiological basis of aggression in zebrafish. To do so, we applied a targeted approach, profiling the expression of 40 genes to identify neurological signalling pathways associated with the production of agonistic behaviors. This was achieved by comparing the brains of zebrafish of different social ranks that differed in their level of aggressiveness. Within this experiment, we included comparisons of different regions of the brain, explored possible sex-related differences and quantified temporal changes in the patterns of gene expression associated with changes in behaviour. Our integrated approach enabled us to link differences in the expression of cascades of genes associated with a wide range of neurological signalling pathways with differences in the levels of aggression. We then performed specific pharmacological manipulations on some of the neurological pathways highlighted as potentially of importance in controlling aggression. These manipulations tested for the functional involvement, and possibly sex-specific roles, of these pathways in aggression in zebrafish.

## Results/Discussion

**Figure 1:**
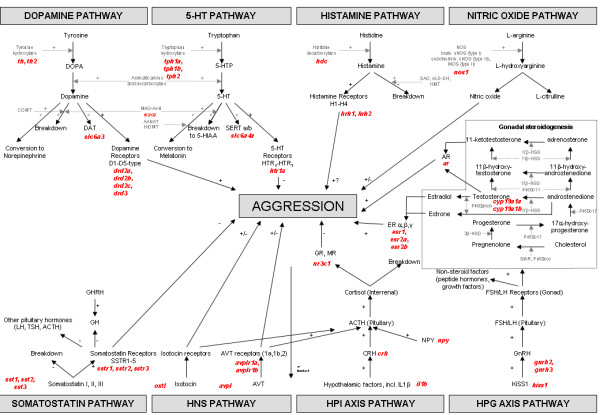
**Simplified schematic of regulatory neurological pathways of aggression in mammals targeted for this study in fish**. Study genes are highlighted in red. Abbreviations: 5-HIAA, 5-hydroindoleacetic acid; 5-HT, 5-hydroxytryptamine (serotonin); 5-HTP, 5-hydroxytryptophan; AANAT, arylalkylamine N-acetyltransferase; ACTH, adrenocorticotropic hormone; ALD-DH, acetaldehyde dehydrogenase; AR, androgen receptor; AVT, arginine vasotocin; COMT, catechol-*O*-methyl transferase; CRH, corticotrophin releasing hormone; DAO, diamine oxidase; DAT, dopamine transporter; DOPA, dihydroxyphenylalanine; ER, estrogen receptor; FSH, follicle-stimulating hormone; GH, growth hormone; GHRH, growth hormone releasing hormone; GnRH, gonadotropin releasing hormone; GR, glucocorticoid receptor; HIOMT, hydroxyindole-O-methyltransferase; HMT, histamine-N-methyltransferase; HNS, hypothalamo-neurohypophysial system; HPI, hypothalamo-pituitary-interrrenal; HPG, hypothalamo-pituitary-gonadal; IL1β, interleukin 1β; LH, luteinizing hormone; MAO, monoamine oxidase; MR, mineralocorticoid receptor; NOS, nitric oxide synthase; NPY, neuropeptide Y; SERT, 5-HT transporter; TSH, thyrotropin-stimulating hormone. Plus (+) and negative (-) symbols indicate the proposed action of a gene/neurotransmitter/pathway on aggression (i.e. stimulatory or inhibitory, respectively).

In this study, we applied gene expression profiling combined with behavioural analysis on individuals in small social groups to initiate an understanding of the neurophysiological basis of aggression in the zebrafish, our experimental model. For this work, 40 genes were selected based on their reported roles in aggression in mammals. These genes spanned eight interrelated neurological pathways: the hypothalamo-neurohypophysial-system (HNS), serotonin (5-HT), somatostatin, dopamine, histamine, nitric oxide, hypothalamo-pituitary-interrenal (HPI) and hypothalamo-pituitary-gonadal (HPG) pathways (Fig. 1). The expression of these genes in the brains of males and females was then compared between dominant and subordinate fish since they displayed different levels of aggressive behaviour (high and low, respectively. We subsequently performed pharmacological manipulations on dominant males and/or females, administering specific agents (known to be effective in mammals) to agonise, antagonise, or inhibit particular receptors or transporters within the neurological pathways identified. We then quantified the outcomes on aggression compared with sham administrations to assess possible roles of those pathways in the regulation of the aggressive behaviours characterised.

### Differences in aggressiveness between social ranks and sexes

**Figure 2:**
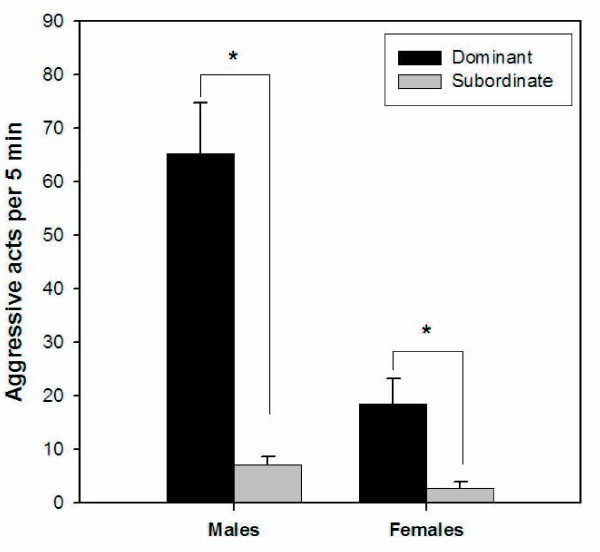
**Correlation between social rank and aggressiveness in male and female zebrafish**. The number of aggressive acts performed by each fish towards the other fish of the same sex in colonies with 2 males and 2 females (n = 16 fish per group) was quantified during a 5 min observation on the day of the sampling. Statistically significant differences in aggression were observed between social ranks for both sexes and between sexes (*P *< 0.001; Two way ANOVA followed by Holm-Sidak post hoc test).

As expected, dominant fish, as quantified by summed frequencies of the specific behaviours of chasing, sparring and repelling, were more aggressive than subordinate fish (Two-way ANOVA: *F*_1,58 _= 45.769; *P *< 0.001; Fig. 2), in accordance with that described previously for this species [22,24]. Males were more aggressive than females (Two-way ANOVA: *F*_1,58 _= 21.967; *P *< 0.001), although the effect of sex on aggression depended on social rank (Two-way ANOVA: *F*_1,58 _= 14.984; *P *< 0.001): dominant males were more aggressive than dominant females but there was no difference in aggression between subordinate males and females (Fig. 2). These data are in keeping with previous findings for this strain of zebrafish [24]. In contrast, Moretz et al. [25] observed no sex differences in the aggression of zebrafish. However, their study used a different measure of aggression (biting to a mirror stimulus) and different strains of zebrafish; in their studies, the choice of zebrafish strain was shown to have a significant influence on their behaviour results [25].

The body sizes (wet weights and fork lengths) of the fish used in the study were: dominant males, 0.374 ± 0.009 g vs. subordinate males 0.331 ± 0.102 g and 31.84 ± 0.32 mm vs. 30.20 ± 0.37 mm; dominant females 0.444 ± 0.015 g vs. subordinate females 0.361 ± 0.047 g and 32.27 ± 0.50 vs. 30.2 ± 0.37 mm, and dominants were significantly larger than subordinates [see [26]]. In the zebrafish used in this study, dominant males also sired more offspring (quantified through parentage analyses using DNA microsatellites on the resulting embryos [24]). This finding was in accordance with that shown previously in competitive breeding scenarios [23]. Dominant females used in this study also had a higher breeding success with the dominant males compared with their subordinates [24]. In our previous work, we have further shown that a higher social rank is correlated with an enhanced general health (e.g. immune and stress status [26]).

### Mapping regions of the brain associated with aggression

**Figure 3:**
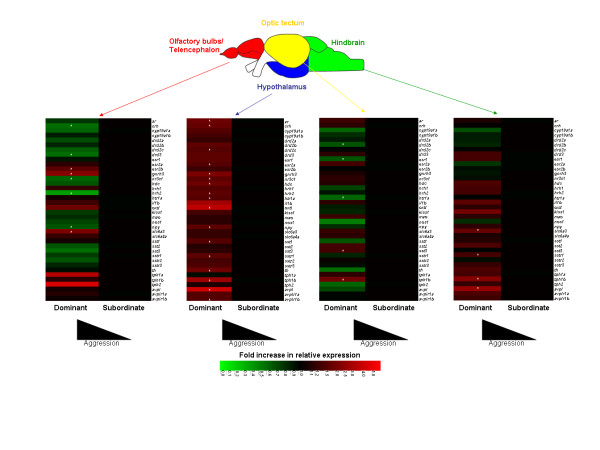
**Regions of the brain associated with differences in aggressiveness in zebrafish**. Heat maps showing all consistently expressed genes in four regions of the brain studied in dominant males (high aggression; n = 8) compared to subordinate males (low aggression; n = 8) sampled on day 1 of social interaction. Genes found to be differentially expressed (*P *< 0.05) are denoted with an asterisk.

We first determined regions of the brain associated with aggressiveness in zebrafish. This was achieved using males, based on their greater aggression, and greater differential in aggression between social ranks, than observed in females. We determined gene expression profiles in four regions of the brain in dominant compared with subordinate males, identified as such based on behavioural observations of aggression on day 1 of the social interaction experiment. In doing so, we observed marked differences with a total of 34 differentially expressed genes across the different brain regions (Fig. 3; for fold-differences and *P *values see Additional File 1). While this number of differentially-expressed genes was relatively large, for the majority of the genes the significance values were between 0.05-0.01. These high values may reflect variability in gene expression between fish, which may have resulted from slight differences in the macroscopic brain dissection process between fish, and/or the modest sample size we used for our analyses (n = 8 fish per group). Furthermore, had we established the relative aggression levels of all the fish used in the study and accounted for these in the statistical analyses of the gene expression data (rather than those between the four fish within each colony only), we may have been able to control for some of the variation observed in gene expression levels.

We observed considerable differences in the specific genes associated with social rank for the different regions of the brain. The greatest differences between social ranks, based on counts of differentially-expressed genes for the chosen set of 40 genes, occurred in the hypothalamus and telencephalon. Here, 17 genes and 8 genes, respectively, were differentially-expressed (Fig. 3). In these regions, the mean fold changes in expression for all genes in dominants compared to subordinates were also the highest (1.71- and 1.26-fold changes, compared to 1.14 and 0.98-fold changes in the hindbrain and optic tectum). This finding in zebrafish is consistent with that occurring in mammals. In mammals, various forebrain structures play key roles in aggression, including the amygdala and hippocampus within the neocortex [6,27], structures that are believed to be homologous to the medial and lateral telencephalon, respectively, in fish [28]. The hypothalamus is also an area associated with the control of aggression in mammals. In its intermediate regions it contains the 'attack area,' which mediates aggressive attacks partially via forebrain stimulation [29], and various neuroendocrine nuclei that regulate pituitary hormone release, for example the preoptic area (POA) located at its border with the ventral telencephalon [6].

We observed intriguing differences in the direction of regulation (i.e. up- or down-regulation) of the differentially-expressed genes for different regions of the brain. In the hypothalamus and hindbrain, all of the genes were overexpressed in dominant individuals (Fig. 3.). In contrast, in the telencephalon and optic tectum, approximately equal numbers of the genes were overexpressed in dominant and subordinate individuals (Fig. 3). Despite the fact that we used a targeted approach, the high number of genes overexpressed in subordinates in the telencephalon is similar to that observed in the analogous region of the brain (the neocortex) in rats. Here, in an array analysis (containing 1178 brain-associated genes), 18 genes were overexpressed in submissive rats but only one gene was overexpressed in dominant rats [30]. The specific genes associated with dominance in the zebrafish varied between brain regions. Only five genes showed the same expression pattern in more than one brain region (*gnrh3, slc6a3*, *sstr1*, *tph1b*, *avpl*; Fig. 3). In contrast, other genes had opposite patterns of expression according to social rank between different regions of the brain (*htr1a*, *hrh2*, *crh*, *nr3c1, npy*; Fig. 3). These data highlight that the use of whole brains for investigating the molecular underpinnings of social behaviours (as in previous studies in fish [31,32]) may lead to inaccurate interpretations. Our work has shown that regional dissection of the brains of zebrafish is a viable approach, even if it may ideally necessitate a higher sample size. Of course, the application of micropunch or laser-capture microdissection technologies to zebrafish would further enhance the anatomical resolution, but these approaches come with additional technical limitations, such as the need for RNA amplification.

### Neurological pathways associated with aggression

Given that we identified the hypothalamus and telencephalon as the regions of the brain where the greatest number of differentially-expressed genes occurred relating to aggressiveness (based on our chosen gene set), these regions were selected for our more focused analyses. The genes differentially expressed in these brain regions relative to social rank, together with their associated functional pathways, are shown in Table 1. This list of genes represented seven functional pathways and suggested a multi-factorial control of aggression in zebrafish. This situation would be in accordance with that suggested to occur for aggression in birds and mammals [33,34]. It is possible that, given the multiple physiological roles of some of these genes including in wider physiological processes other than behavior, some of these differences observed in gene expression levels according to social status may relate to other physiological differences between dominant and subordinate zebrafish, for example, in motor function, metabolism or reproduction. Nevertheless, the commonality of the pathways and genes identified in the zebrafish suggests a high degree of conservation in the control of aggression between fish and mammals and this is a novel finding.

**Table 1:** Genes associated (*P *< 0.05) with aggressiveness and representative neurological pathways in male zebrafish, based on analyses performed using data from hypothalamus and telencephalon of dominant (high aggression) and subordinate (low aggression) males sampled on day 1 of interaction.

Region of brain	GenBank accession no.	Gene	Description	Pathway	Fold-increase in dominants (mean ± SEM)	*P*-value
						

**Genes overexpressed in dominants**						

Hypothalamus	NM_178293	*avpl*	*arginine vasopressin-like*	HNS	4.31 ± 1.60	0.038

	XM_695195	*avplr1b*	*arginine vasopressin-like receptor 1b*	HNS	1.56 ± 0.18	0.020

	NM_178291	*oxtl*	*oxytocin-like*	HNS	3.77 ± 1.01	0.023

	NM_001001843	*tph1b*	*tryptophan hydroxylase 1b*	5-HT	2.98 ± 0.69	0.017

	EH441641	*htr1a*	*5-hydroxytryptamine (serotonin) receptor 1A*	5-HT	1.59 ± 0.21	0.027

	AF435965	*sst1*	*somatostatin 1*	Somatostatin	1.56 ± 0.21	0.048

	XM_691574	*sstr1*	*somatostatin receptor 1*	Somatostatin	1.67 ± 0.22	0.019

	AF075384	*th*	*tyrosine hydroxylase*	Dopamine	1.94 ± 0.33	0.021

	AY333792	*drd2c*	*dopamine receptor d2c*	Dopamine	1.58 ± 0.22	0.044

	EF150846	*hdc*	*histidine decarboxylase*	Histamine	1.88 ± 0.17	0.003

	NM_001045338	*hrh2*	*histamine receptor h2*	Histamine	1.73 ± 0.28	0.046

	NM_001007379	*crh*	*corticotropin releasing hormone*	HPI	1.75 ± 0.26	0.033

	EF567112	*nr3c1*	*nuclear receptor subfamily 3, group C, member 1 (glucocorticoid receptor)*	HPI	1.66 ± 0.25	0.035

	NM_131074	*npy*	*neuropeptide y*	HPI	1.67 ± 0.19	0.024

	AJ304429	*gnrh3*	*gonadotropin-releasing hormone 3*	HPG	2.16 ± 0.34	0.015

	NM_180966	*esr2a*	*estrogen receptor 2a*	HPG	1.52 ± 0.18	0.030

	NM_001083123	*ar*	*androgen receptor*	HPG	1.96 ± 0.30	0.010

Telencephalon	NM_131755	*slc6a3*	*solute carrier family 6 (neurotransmitter transporter, dopamine), member 3*	Dopamine	2.25 ± 0.45	0.029

	AJ304429	*gnrh3*	*gonadotropin-releasing hormone 3*	HPG	2.37 ± 0.50	0.025

	AJ414566	*esr2b*	*estrogen receptor 2b*	HPG	1.63 ± 0.21	0.045

**Genes overexpressed in subordinates**						

Telencephalon	NM_183067	*drd3*	*dopamine receptor d3*	Dopamine	0.56 ± 0.09	0.041

	NM_001045338	*hrh2*	*histamine receptor h2*	Histamine	0.38 ± 0.05	0.010

	NM_001007379	*crh*	*corticotropin releasing hormone*	HPI	0.56 ± 0.07	0.044

	EF567112	*nr3c1*	*nuclear receptor subfamily 3, group C, member 1 (glucocorticoid receptor)*	HPI	0.62 ± 0.05	0.023

	NM_131074	*npy*	*neuropeptide y*	HPI	0.68 ± 0.08	0.029

Interestingly, the differences in expression of the various genes studied was relatively low between the behavioural phenotypes (for the majority of genes, below 2-fold; Table 1). This may indicate that functional of the zebrafish brain with respect to aggressiveness is driven by relatively small differences in individual genes that become of great significance when integrated together into functional gene networks. Of course, it is also possible that there is an amplification of the signals from these relatively small differences in gene expression through post translational events. Other studies on gene expression in the brain have shown that profound differences in phenotype are often associated with only small changes in gene expression [12]. The relatively low level changes in gene expression in the brain is, in part, suggested to be the result of a tight homeostatic control in the nervous system [12].

**Figure 4:**
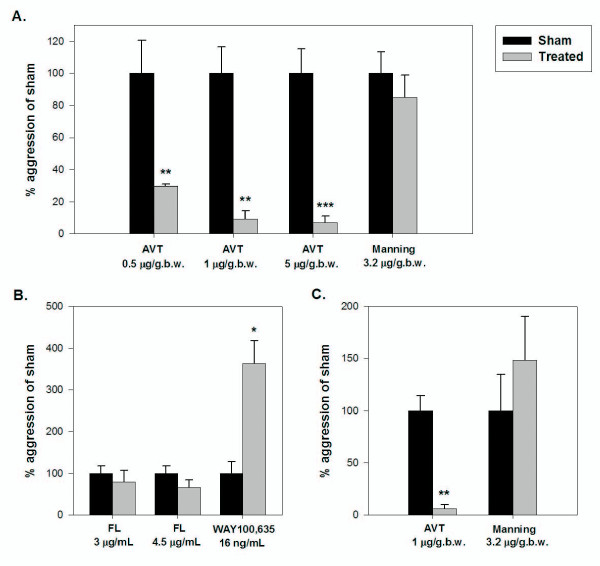
**Aggression of zebrafish following pharmacological manipulations of various nodes within neurological pathways proposed to regulate aggressive behaviour**. A. The hypothalamo-neurohypophysial system (HNS) pathway in males. B. The serotonin (5-HT) pathway in males. C. The HNS pathway in females. Specific agents known to agonise or antagonise particular receptors or inhibit transporters within the identified axes were administered and the effect on aggression determined (treated) compared to a sham administration (sham) with n = 6-9 fish. AVT, arginine vasotocin acetate salt; Manning, Manning compound; FL, fluoxetine HCl; WAY100,635, WAY100,635 maleate salt. Significant changes in aggression (number of aggressive acts per 5 min observation) are indicated by asterisks (*P < 0.05; **P < 0.01; ***P < 0.001).

*avpl *(*arginine vasopressin-like*) showed the greatest difference in expression between dominant and subordinate males (4.3-fold overexpression in dominant males; Table 1). This was consistent with an observation based on a microarray analysis (with 3.6K unique sequences) of whole brains in the cichlid fish *Astatotilapia burtoni *[32] and provides strong support for a role of this peptide in aggression in the zebrafish. The product of the *avpl *gene, arginine vasotocin (AVT), and its mammalian homologue arginine vasopressin (AVP), have well established roles in aggression and social position across vertebrate taxa [35]. To test this hypothesis in our zebrafish model, we administered AVT at three doses intraperitoneally (i.p.) to dominant males and observed a highly significant reduction in their aggression (to 7-30% of sham; paired *t*-tests: *t*_6 _= 4.568 and *P *= 0.004; *t*_7 _= 5.227 and *P *= 0.001; *t*_8 _= 7.286 and *P *< 0.001; for 0.5, 1, and 5 μg/g.b.w. doses, respectively; Fig. 4A). These inhibitory effects of AVT on aggression in zebrafish contradict those for some other fish species [36,37]. This may be because AVT increases aggression in non-territorial (colonial) species and decreases it in territorial species [35], however. Even within a single species, AVT can have opposite effects between behavioural phenotypes; AVT increases aggression in non-territorial males but decreases aggression in territorial males [38]. This may also be the case in zebrafish, with AVT having an opposite effect on dominant (territorial) versus subordinate (non-territorial) individuals and this should be a focus for future studies on the role of AVT in aggression in zebrafish.

Interestingly, of the two genes we quantified encoding AVT's receptors, only *avplr1b *was differentially expressed between dominant and subordinate males (Table 1). In rodents, the 1b receptor plays a key role in regulating the actions of AVT on aggression [39]. However, the role of this receptor in aggression has, to date, not been studied in non-mammalian vertebrates. We were unable to source a receptor agonist/antagonist specific to the 1b receptor to test for functionality in zebrafish. We were able to test for an effect of the 1a receptor using the specific AVT 1a receptor antagonist Manning compound, however. Following i.p. injection of Manning compound into dominant males we observed no significant difference in aggression (paired *t*-test: *t*_7 _= 1.762; *P *= 0.121; Fig. 4A). The dose of Manning compound we adopted for this work in zebrafish has been shown to inhibit aggression in some other fish species [37,38], although not in all [40].

We also observed *oxtl (oxytocin-like)*, which encodes isotocin (the fish homologue of oxytocin), to be overexpressed in dominant males (Table 1). Isotocin has been shown to have no effect on aggression, and even stimulated sociality, in other fish species [37,41]. This is a response well established for oxytocin in mammals [42]. Nevertheless, it is believed that oxytocin's prosocial role (particularly in pair-bond formation) could stimulate aggression towards intruders for the purpose of maintaining monogamy [43], as shown in female Wistar rats [44] and mandarin voles [45].

Within the 5-HT pathway, the genes encoding 5-HT's synthesising enzyme (*tph*) and one of its receptors (*htr1a*) were overexpressed in dominant versus subordinate males (3-fold and 1.6-fold, respectively; Table 1). To test for a possible role for 5-HT in aggression in zebrafish (as found in birds and mammals), we manipulated the 5-HT system of dominant males using fluoxetine and WAY100,635. Fluoxetine increases synaptic 5-HT levels by blocking its reuptake via the 5-HT transporter (SERT), and WAY100,635 is an antagonist of the HTR_1A_. Counts of aggression in dominant males decreased to 66-79% of the sham-treated level following fluoxetine treatment (Fig. 4B). However, these apparent suppressions were not statistically significant (paired *t*-tests: *t*_4 _= 1.118 and *P *= 0.326; and *t*_5 _= 2.371 and *P *= 0.064, respectively for the 3 and 4.5 μg/L concentrations). This lack of effect of fluoxetine may relate to our choice of a 1 hour exposure duration (to enable the behavioural observations to be undertaken in the morning when levels of aggression are highest in zebrafish [24]) rather than for the longer duration of 3 hours that has been shown to decrease aggression in a previous study on *Betta splendens *[46]. Males exposed to WAY100,635, in contrast, showed greater aggression (3.6-fold; paired *t*-test: *t*_7 _= -2.643; *P *= 0.033; Fig. 4B). These findings of the pharmacological manipulations are consistent with an inhibitory role for 5-HT in aggression in zebrafish, as implied in studies with these compounds in other fish [46-48].

These results of our pharmacological manipulations of the HNS and 5-HT systems strongly support a utility of gene expression profiling for predicting neurological pathways regulating aggression in the zebrafish. Not all gene responses conformed to the functional responses, however. We might, for example, have expected that in the less aggressive subordinate males there would have been an increased expression of HNS and 5-HT pathway genes to inhibit their aggression (to comply with the inhibitory roles of AVT and 5-HT demonstrated through the manipulations), but this was not the case. Also, expression levels of *sst1*, encoding the somatostatin peptide, and *sstr1*, encoding one of its receptors, were higher in dominant males (Table 1) but somatostatin inhibited aggression in the fish *A. burtoni *[49]. We are unable to explain these discrepancies at this time. Analyses of the protein levels of the target gene products (e.g. TPH) and/or transcript or protein localisation studies, via in situ hybridisation or immunohistochemical methodologies, may give possible further insight into this in the future. Alternatively, some neurotransmitters are known to have complex and multiple roles, with both stimulatory and inhibitory effects on aggression in the same species. For example, in mammals, 5-HT (which normally inhibits aggression) is actually released during initial aggression in individuals that subsequently become dominant, but at this time it loses its inhibitory function [50,51].

Enhanced expression of two dopamine pathway genes (*th *and *slc6a3*, which encode the enzyme tyrosine hydroxylase and the dopamine transporter (DAT), responsible for dopamine synthesis and re-uptake, respectively, the latter of which ultimately terminates dopaminergic activity), were associated with higher aggression (Table 1). This is a novel finding for fish and is consistent with the stimulatory role of dopamine, and its associated genes, in aggression and dominance in mammals [52-54]. In contrast to *th *and *slc6a3*, *drd3 *was overexpressed in subordinates (Table 1). This finding was consistent with that occurring in the analogous region of the brain in rats [30] and another new finding in non-mammalian vertebrates. In mammals, the D3 receptor, which is highly expressed in limbic areas of the brain, functions in motor behaviour and emotional reactivity (e.g. escape) within social situations [55]. The D2 receptor is known to play a key role in modulating the stimulatory effects of dopamine on aggression in mammals [54]. Our data, therefore, suggest opposing behavioural effects of the D3 and D2 receptors, as proposed in mammals [56].

In the HPG axis, we observed elevated expression of the genes encoding the androgen receptor (AR) and estrogen receptor (ER) β subtypes (*ar*, *esr2a*, *esr2b*) in dominant males (Table 1). This supports an enhanced responsiveness of the brains of dominant fish to sex steroids, which have stimulatory roles in aggression/dominance [57]. In mammals, ERα is the candidate ER for mediating the actions of estrogens (and androgens, following aromatisation) on aggression [58,59]. ERβ instead may inhibit aggression [60]. Upstream in this pathway, *gnrh3 *(the protein product of which stimulates sex steroid production) had 2.2-2.4-fold greater expression in dominant males compared with subordinates (Table 1). This difference is analogous to findings in *A. burtoni *[61], but is perhaps surprising given that a subordinate rank does not prevent reproduction in zebrafish [23,24], as in that species.

In the histamine pathway, we observed overexpression of the genes encoding the histamine-producing enzyme L-histamine decarboxylase (*hdc*) and the H_2 _receptor (*hrh2*) in dominant males (Table 1). Histamine has been shown to modulate other behaviours in the zebrafish [62,63]. However, a possible role for histamine in aggression has not yet been put forward in zebrafish, or indeed in any non-mammalian vertebrate, so this is another novel finding. In mammals, histamine facilitates aggression, via H_1 _receptors, probably by inhibiting 5-HT [64,65].

In the HPI axis (homologous to the HPA axis in mammals), *crh*, *nr3c1 *(formerly known as *glucocorticoid receptor*), and *npy *expression were associated with aggressiveness. However, these genes had opposite expression profiles in the hypothalamus compared with the telencephalon (Table 1). All individuals show a stress response upon social interaction [51]. However, a lower stress responsiveness has been associated with an increased likelihood of individuals becoming dominant [66] and, in humans, with high levels and/or abnormal forms of aggression [67]. Corticosteroids, which remain elevated long-term in subordinates, suppress aggression via elevation of brain 5-HT [68]. The overexpression we observed for *crh *and *npy *in the telencephalon of subordinate males likely represents an enhanced stress response in these fish compared with dominants. This is because these peptides from the POA in this region ultimately induce corticosteroid release. Both CRH and NPY influence aggression in mammals [18,69-71] and/or fish [72], although the effects of CRH varied depending on the dose given and the region of the brain to which it was administered. This may explain the spatial differences we observed in *crh *expression.

### Sex-related differences in genes associated with aggression

Female zebrafish, as well as male, show territorial aggression [24]. A further dimension to our analyses was, therefore, the assessment of possible sex-related differences in neuroregulatory pathways of aggression using females sampled on the same days as the males. Little study has been carried out in this regard, which is surprising given that female aggression, including territorial aggression, is frequently encountered in animals and is an important component of mating and social systems [73,74]. The ability to study social rank in both sexes, and thus partition the effects of aggression and sex, is a powerful experimental feature of the zebrafish model system. The zebrafish is also of particular interest for comparative studies on sex differences in vertebrates due to the absence of a defined sex determination mechanism (e.g. SRY) or discernible sex chromosomes.

**Figure 5:**
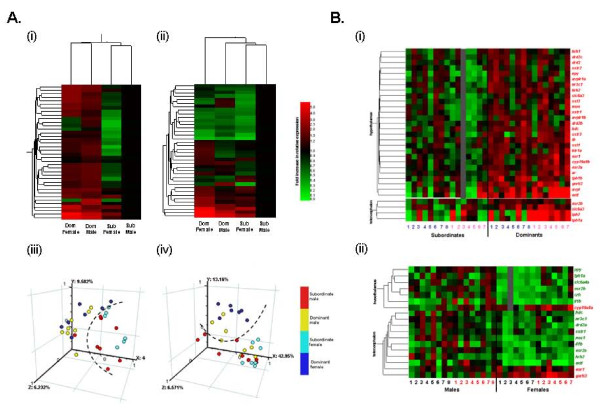
**Comparisons of the roles of sex and social rank as determinants of the brain gene expression profiles**. A. Hierarchical cluster analysis of all consistently expressed genes in (i) hypothalamus and (ii) telencephalon of dominant (high aggression) and subordinate (low aggression) males and females (n = 7-8 fish per group) and the corresponding PCAs performed on the profiles of the same individual fish in (iii) hypothalamus and (iv) telencephalon) (the dashed lines were drawn arbitrarily to aid the visualisation of clustering of the samples). B. Hierarchical cluster analysis of genes identified as differentially expressed (*P *< 0.05) between (i) dominants (high aggression) and subordinates (low aggression) irrespective of their sex, and (ii) between males and females irrespective of their social rank. Fish are shown individually. In (i), individual male fish are indicated by numbers in blue and individual female fish by numbers in pink (all genes were overexpressed in dominant fish). In (ii), individual dominant fish are indicated by numbers in red and individual subordinate fish by numbers in black. In (i)-(ii), genes shown in red were overexpressed in females while genes shown in green were overexpressed in males. Grey colouration indicates no data.

To probe for the main neurological determinants of aggression in zebrafish, based on expression of the targeted genes, we analysed our entire dataset (both males and females for all consistently-expressed genes) using hierarchical clustering and principal component analysis (PCA). The clustering demonstrated that in the hypothalamus, social rank (and, consequently, aggressiveness) was a greater determinant of the expression of the chosen gene set than sex (Fig. 5Ai). In telencephalon, similarly, subordinate males were grouped with subordinate females, rather than with dominant males (Fig. 5Aii). These results were further supported by the PCAs, which generally grouped individual fish according to social rank rather than sex, identifying clear differences between ranks in both regions of the brain, albeit this was most clearly the case in the hypothalamus (Fig. 5Aiii-iv). These data suggest that similar expression profiles may regulate aggression between sexes in zebrafish. In fact, we identified 27 genes in the hypothalamus and 4 genes in the telencephalon that were differentially expressed according to social rank (i.e. always higher in dominants or in subordinates, irrespective of sex; Fig. 5Bi) but only 5-8 genes differentially expressed according to sex (i.e. always higher in males or in females, irrespective of rank) in these brain regions, respectively (Fig. 5Bii).

28 genes were differentially expressed between ranks in females (Table 2). This greater number of genes compared with that in males was surprising given the lower counts of aggressive behaviours recorded for this sex (Fig. 2). Nevertheless, within the sexes, dominants were similarly more aggressive than subordinates (9- and 7-fold in males and females, respectively) (Fig. 2). In females, the greatest number of differentially expressed genes occurred in the hypothalamus (21 genes; Table 2), and these genes belonged to the same neurological pathways observed in males, indicating conservatism in the control of aggression between the sexes in zebrafish. In females, the genes that showed the greatest difference between dominants and subordinates were *tph1a*, *npy *and *avpl*, all of which were more highly expressed in dominants (Table 2). 14 genes were up- or down-regulated in both dominant males and females compared to subordinates in at least one brain region (*avpl*, *avplr1b*, *oxtl*, *th*, *sst1*, *htr1a*, *hrh2*, *nr3c1*, *crh*, *gnrh3*, *esr2a *and *ar *in hypothalamus, and *esr2b*, *slc6a3 *and *gnrh3 *in telencephalon; Tables 1, 2).

**Table 2:** Genes associated (*P *< 0.05) with aggressiveness in female zebrafish, based on analyses performed using data from the hypothalamus and telencephalon of dominant (high aggression) and subordinate (low aggression) females sampled on day 1 of interaction.

Region of brain	GenBank accession no.	Gene	Description	Pathway	Fold-increase in dominants (mean ± SEM)	*P*-value
**Genes overexpressed in dominants**						

Hypothalamus	NM_178293	*avpl*	*arginine vasopressin-like*	HNS	3.89 ± 0.82	0.014

	XM_695195	*avplr1b*	*arginine vasopressin-like receptor 1b*	HNS	3.04 ± 0.82	0.022

	NM_178291	*oxtl*	*oxytocin-like*	HNS	3.19 ± 0.58	0.016

	NM_214795	*tph2*	*tryptophan hydroxylase 2*	5-HT	2.83 ± 0.46	0.005

	EH441641	*htr1a*	*5-hydroxytryptamine (serotonin) receptor 1A*	5-HT	2.89 ± 0.72	0.037

	DQ285098	*slc6a4a*	*solute carrier family 6 (neurotransmitter transporter, serotonin), member 4a*	5-HT	3.44 ± 0.57	0.001

	NM_212827	*maoa*	*monoamine oxidase a*	5-HT	3.52 ± 0.82	0.014

	AF435965	*sst1*	*somatostatin 1*	Somatostatin	1.77 ± 0.28	0.042

	BI473045	*sst3*	*somatostatin 3*	Somatostatin	3.61 ± 1.00	0.002

	AF075384	*th*	*tyrosine hydroxylase*	Dopamine	2.10 ± 0.40	0.038

	NM_131755	*slc6a3*	*solute carrier family 6 (neurotransmitter transporter, dopamine), member 3*	Dopamine	2.56 ± 0.56	0.030

	NM_001045338	*hrh2*	*histamine receptor h2*	Histamine	1.96 ± 0.30	0.029

	NM_131660	*nos1*	*nitric oxide synthase 1 (neuronal)*	Nitric oxide	2.51 ± 0.51	0.022

	NM_001007379	*crh*	*corticotropin releasing hormone*	HPI	1.88 ± 0.29	0.025

	EF567112	*nr3c1*	*nuclear receptor subfamily 3, group C, member 1 (glucocorticoid receptor)*	HPI	2.02 ± 0.38	0.040

	NM_131074	*npy*	*neuropeptide y*	HPI	4.37 ± 0.95	0.002

	AJ304429	*gnrh3*	*gonadotropin-releasing hormone 3*	HPG	2.78 ± 0.67	0.044

	NM_131642	*cyp19a1b*	*cytochrome P450, family 19, subfamily A, polypeptide 1b*	HPG	2.95 ± 0.57	0.001

	AB037185	*esr1*	*estrogen receptor 1*	HPG	3.02 ± 0.80	0.005

	NM_180966	*esr2a*	*estrogen receptor 2a*	HPG	2.80 ± 0.55	0.022

	NM_001083123	*ar*	*androgen receptor*	HPG	2.61 ± 0.52	0.014

Telencephalon	XM_695195	*avplr1b*	*arginine vasopressin-like receptor 1b*	HNS	1.80 ± 0.22	0.031

	AF548566	*tph1a*	*tryptophan hydroxylase 1a*	5-HT	4.61 ± 0.74	0.001

	NM_214795	*tph2*	*tryptophan hydroxylase 2*	5-HT	3.09 ± 0.55	0.016

	BI473045	*sst3*	*somatostatin 3*	Somatostatin	1.92 ± 0.33	0.029

	NM_131755	*slc6a3*	*solute carrier family 6 (neurotransmitter transporter, dopamine), member 3*	Dopamine	2.48 ± 0.48	0.017

	AJ304429	*gnrh3*	*gonadotropin-releasing hormone 3*	HPG	1.87 ± 0.35	0.048

	AJ414566	*esr2b*	*estrogen receptor 2b*	HPG	1.79 ± 0.27	0.029

Increased expression of *avpl *and *avplr1b*, and *avpl *being one of the genes most associated with aggression in females, as occurred in males, is a particularly interesting finding as AVT is best known for its roles in male-specific behaviours [35] and males often have greater numbers/size of AVT neurons [74,75]. Consistent with our results, however, *avpl *was shown to be differentially expressed between social ranks in both sexes in *Neolamprologus pulcher *[76] and, in mice, deletion of the gene for the AVP receptor 1b suppressed aggression in both sexes [77,78]. To test whether AVT also regulates aggression in females, we repeated our manipulations with AVT and Manning compound in females and found identical effects as observed in males. Injection of dominant females with AVT decreased their aggression to 6% of the sham level (paired *t*-test: *t*_5 _= 6.170; *P *= 0.002) while Manning compound had no effect (paired *t*-test: *t*_7 _= -0.264; *P *= 0.799) (Fig. 4C).

We did observe some differences in the expression of genes associated with dominance between the sexes (Tables 1, 2), including an association of some genes with aggression in females only (*tph1a*, *tph2*, *sst3*, *slc6a4a*, *slc6a3*, *nos1*, *mao*, *cyp19a1b*, *esr1*), and some in males only (*tph1b*, *sstr1*, *drd2c*). Our results for the *tph *genes suggest a sex-related difference in the subtypes of the 5-HT synthesising enzyme TPH that may regulate aggression, with the situation in females being more analogous to that in humans [79]. The result for *esr1 *was also interesting given that in mice ERα deletion had opposite effects on aggression between sexes [59,80]. In relation to *nos1*, in mammals neural nitric oxide (the gas product of which acts as a neurotransmitter [81]) inhibits aggression in males [82] but stimulates aggression in females [18,83], again showing conformity in the findings for different sexes in the zebrafish compared with in mammals.

### Temporal changes in gene expression associated with changes in aggression

**Figure 6:**
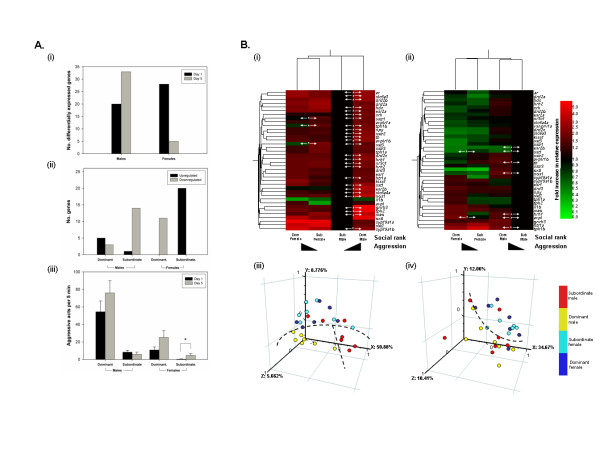
**Temporal changes in gene expression correlated with changes in aggression**. A. Phenotypic anchoring of (i) temporal changes in the total number of genes that were differentially-expressed between social ranks, which differed in aggressiveness (across hypothalamus and telencephalon) and (ii) parallel changes identified in the expression levels of individual genes within ranks over the same time with (iii) temporal changes in aggressiveness of zebrafish between day 1 and day 5. B. Hierarchical cluster analysis of all genes in (i) hypothalamus and (ii) telencephalon of dominant (high aggression) and subordinate (low aggression) males and females and the corresponding PCAs performed on the profiles of the same individual fish (n = 7-8 per group) in (ii) hypothalamus and (iv) telencephalon (the dashed lines drawn arbitrarily indicate clustering of the samples) on day 5. Statistically significant changes (*P *< 0.05) are indicated by an asterisk.

To identify temporal variation in the genes associated with aggression, we compared our data for fish sampled on day 1 of the social interaction experiment with those for equivalent fish sampled after 5 days (Fig. 6). The number of genes differentially expressed according to rank across the two brain regions altered with time, but differed between sexes (Fig. 6Ai). In males, the number of differentially-expressed genes increased from 20 genes on day 1 to 33 genes on day 5 (Fig. 6Ai). The expression levels of 14 genes were reduced in subordinates and 5 genes increased in dominants over this time (Fig. 6Aii; also see Additional Files 2 and 3). In females, however, the number of differentially expressed genes decreased from 28 genes to 5 (Fig. 6Ai). The expression levels of 20 genes increased in subordinates over the 5 days and of 9 genes reduced in dominants (Fig. 6Aii; also see Additional Files 2 and 3). These changes in gene expression aligned closely with phenotypic-level behavioural changes observed in these fish (Fig. 6Aiii). In males, the level of aggressiveness of the dominant male compared with the subordinate increased from 6.5-fold to 12-fold between day 1 and day 5, respectively (Fig. 6Aiii), matching the increased divergence observed in their gene expression profiles. In contrast, in females, the aggressiveness of the dominant female towards the subordinate decreased from 25- fold higher to 5-fold higher (Fig. 6Aiii) and aggression by the subordinate increased substantially (by 11-fold) over this time (Mann-Whitney rank sum test; *t*_14 _= 36; *P *= 0.021). Again these changes in behaviour of the female zebrafish aligned well with the patterns of gene expression with the reduction in differences (number of differentially expressed genes) observed between female ranks and increased expression of many 'aggression' genes in the hypothalamus in subordinate females.

The individual genes differentially expressed between dominant and subordinate fish on day 5 are shown in Fig. 6Bi-ii. When we compared these results with those for day 1 (Table 1), we found that in the hypothalamus of males (Fig. 6Bi), 88% of the genes overexpressed in dominants on day 1 were also overexpressed on day 5: only the overexpressions of *avpl *and *oxtl *were specific to dominants in day 1, although 11 genes were specific to dominant males on day 5 (*tph2*, *sst3*, *sstr2*, *slc6a4a*, *slc6a3*, *nos1*, *mao*, *hrh1*, *esr2b*, *drd2b*, *cyp19a1b*). In males (Fig. 6Bii), however, the genes differentially expressed between the dominants and subordinates in the telecephalon on day 5 were entirely different to those on day 1. Similarly, in females, none of the genes that were differentially expressed on day 1 were also differentially expressed in the same direction on day 5 (with the exception of *sst3 *which was regulated in an opposite direction on the two days (Fig. 6Bi-ii)). *avpl *and *sst1 *in the telencephalon were the only genes that showed the same patterns of expression in dominants versus subordinates in both sexes on day 5. When the data for both sampling days were combined, only *esr1 *was differentially expressed between social ranks in females. This may be a strong candidate for further studies on sex differences in the control of aggression in zebrafish.

Hierarchical clustering and PCAs of these data on day 5 (Fig. 6B), analogous to those performed on the data for day 1 (Fig. 5A), demonstrated that social rank was no longer the main determinant of the expression of the study gene set; rather sex was more important. In the cluster analyses (Fig. 6Bi-ii), the similarity in the gene expression profiles of the dominant and subordinate females (particularly in the hypothalamus) resulted in their placement together in a single condition cluster, rather than with the males of the same social ranks. Similarly, the PCAs (Fig. 6Biii-iv) did not identify any distinct differences between the dominant and subordinate females, thus grouped individuals according to sex. In the hypothalamus (Fig. 6Biii) clear differences were, nonetheless, identified between the dominant and subordinate males. Additionally, the individual males appeared more segregated by rank than observed on day 1 (Fig. 5iii), again aligning with the increased differential in aggression between male ranks by day 5.

## Conclusions

Aggression is an evolutionary conserved behaviour with implications for human and animal societies and unravelling its neurophysiological basis has many potential benefits. We identified numerous, including novel, candidate neurological genes and pathways associated with aggression in fish, including those possibly conferring sex related differences. For some of these pathways, we further directly linked the pathways with functional responses, albeit our manipulation experiments were limited and we did not, for example, include any determination of dose/concentration-responses. The conservation of many aspects of aggression neurophysiology in zebrafish and mammals supports the use of this fish model in aggression research generally. Further work on aggression using the zebrafish model should establish whether different forms of aggression, the same forms of aggression in different social contexts, and aggression by dominant/territorial compared to subordinate/non-territorial fish are regulated by similar mechanisms. Additionally, further work needs to investigate the inter linkages between aggression control pathways and develop a greater understanding of sex related differences.

## Methods

### Zebrafish stocks

The zebrafish used were bred in-house from the Wild Indian Karyotype (WIK) strain. Fish were maintained at 28 ± 1°C with a constant photoperiod of 12 h light: 12 h dark (08.30-20.30) and an artificial dawn/dusk transition of 30 min. The tank water was reconstituted reverse osmosis-filtered mains water supplied on a flow-through system (2 L/hour). Prior to the experiments, fish were maintained in large mixed-sex holding tanks. The experimental tanks used were 300 mm × 300 mm × 300 mm with a working volume of 18 L and contained a central spawning site, consisting of glass marbles with an artificial weed. Opaque dividers prevented visual interactions between fish in neighbouring tanks. Fish were fed to satiation twice daily with freshly hatched *Artemia *nauplii (ZM Ltd., Hampshire, UK) and TetraMin tropical flake food (Tetra Werke, Melle, Germany). All animal-use procedures were carried out ethically according to UK Home Office guidelines.

### Social interaction study: experiment design and sampling

Immediately prior to the artificial dusk on day zero, 64 fish (32 males and 32 females) were assigned randomly into 16 experimental tanks, with two males and two females per tank, and the fish were allowed to acclimate overnight (such that the fish recovered from any handling stress encountered, resumed 'normal' behaviour and developed a dominance hierarchy). The following morning, beginning at 08.30 am, 8 of the tanks were videotaped for 10 min using an Ikegami ICD-848P digital colour-black/white video camera (Ikegami Co. Ltd., Tsushinki, Japan) coupled to a Sony HDV 1080i digital HD videocassette recorder. Dominance/subordinance was assigned for each fish (based on territoriality - occupation and defence of the spawning site), and the fish were sacrificed by terminal anaesthesia (benzocaine; Sigma, UK). Four distinct brain regions - the telencephalon (including the olfactory bulbs and POA), hypothalamus, optic tectum, and hindbrain (medulla oblongata and cerebellum) - were dissected from each fish, snap-frozen in liquid nitrogen, and stored at -80°C until processing. For the remaining 8 tanks, the same procedure was conducted on day 5 of the study. Based on observations of behaviour, a dominant-subordinate relationship formed between the two males and between the two females in all, but one of the colonies. In the exception, no obvious hierarchy developed between the two females, and this colony was, therefore, excluded from further analyses.

### Behavioural analyses

HDV files containing behavioural recordings were streamlined to AV1 files using Adobe Premier, and viewed at half speed with Windows Media Player. Aggressive behaviours ('chase', 'spar', 'repel' [24]) performed by each fish to its dominant/subordinate were counted for 5 min.

### RNA extraction and reverse transcription

RNA was extracted separately from each of the four brain regions of each individual fish using the RNeasy Micro Kit (Qiagen, Crawley, UK), which included a DNase treatment, and RNA quantity/quality was verified by Nanodrop ND-1000 spectrophotometer (Nanodrop Technologies, Inc., Wilmington, DE, USA). For each individual tissue sample (4 per fish, giving a total of 256 samples), cDNA was synthesized from the total amount of RNA obtained (approximately 20-100 ng) using random hexamers (MWG-Biotech, Ebersburg, Germany) and M-MLV reverse transcriptase (Promega, Southampton, UK).

### Real-time PCR

A suite of 40 genes involved in HNS, 5-HT, somatostatin, dopamine, histamine, nitric oxide, HPI axis, and HPG axis pathways were selected for study in zebrafish based on the literature for studies on aggression in mammals (Fig. 1). For several of the target genes (*avplr1b*, *sstr1*, *sstr2*, *sstr3*), annotated cDNA sequences were not available for zebrafish. For these genes, cDNA sequences were identified by querying the zebrafish genome database with the sequences for these cDNAs in other fish species using the BLAST algorithm. Hits obtained from the BLASTs were then screened for conserved motifs specific to each transcript via multiple sequence alignment (ClustalW). Primers specific for the target genes were designed with Beacon Designer 3.0 (Premier Biosoft International, Palo Alto, CA, USA) and assays optimised and validated for real-time PCR using SYBR Green chemistry, as described previously [84] (also see Additional File 4). Real-time PCR was conducted in triplicate for each individual sample (n = 256) on 1:3-diluted cDNA with the iCycler iQ Real-time Detection System (Bio-Rad Laboratories Inc., Hercules, CA) as previously described [84] but with the reaction volume scaled-down to 15 μL. Relative expression levels (gene of interest: 'housekeeping' gene) were determined using the arithmetic comparative 2^-ΔΔCt ^method with efficiency correction as previously described [84]. *ribosomal protein L8 *(*rpl8*) was used for relative quantification because its expression was unaffected by social rank, sex, or duration of interaction. For further details of the real-time PCR assays and methodologies used, see Additional File 4.

### Pharmacological manipulations

Pharmacological agents known to manipulate specific nodes within the HNS and 5-HT pathways in fish/mammals were administered to dominant males or females via i.p. injection or immersion (epithelial uptake). For all manipulations, a repeated-measures design was used whereby the same fish were tested under both sham and treated conditions. The order of treatments was randomised for each fish within each experiment. On the morning of the day before the start of each experiment, 24 fish were randomly allocated to 12 tanks with 2 fish per tank and a very small section of caudal fin was removed from one of the fish from each tank to permit identification of individual fish (this procedure did not affect a fish's social rank/behaviour and the tail grew back completely after 7-10 days). That afternoon, social rank was assigned for each fish and the tanks in which the most stable hierarchies had developed were selected for the drug manipulations (minimum n = 6, maximum n = 9). The following morning, the dominant fish from each tank was removed for experimental treatment. When immersion was used as the route of drug delivery, a stock solution of the chemical was prepared in aerated aquarium water and fish exposed to either this (treated) or untreated aquarium water (sham) in a glass beaker (50 mL volume) at 28°C for 1 h. When injection was used as the route of drug delivery, the chemical was dissolved in 0.9% saline and fish injected into the i.p. cavity with either this (treated) or saline alone (sham), using a 0.3 mL 30G needle syringe (BD Micro-Fine U-100 insulin syringe) with a 10 μl injection volume. Following a 40 min recovery period for the fish in their respective tanks, behavioural analyses were conducted blind in duplicate. In these analyses, the total number of aggressive acts performed by the dominant fish towards the subordinate was quantified over a period of 5 min. All fish were given 24-48 hours (based on pilot studies) recovery prior to the subsequent treatment within an experiment. For the HNS axis, we tested the natural AVT peptide, AVT acetate salt (Sigma V0130), which was administered by i.p. injection at doses of 0.5, 1, and 5 μg/g.b.w. to males and 1 μg/g.b.w. to females, and the specific AVT receptor 1a antagonist Manning compound ([β-Mercapto-β,β-cyclopentamethylenepropionyl^1^, O-me-Tyr^2^, Arg^8^]-Vasopressin [85]; Sigma V2255), also administered via i.p. injection but at a dose of 3.2 μg/g.b.w. to both males and females. For the 5-HT axis, we tested the HTR_1A _antagonist, WAY 100,635 maleate salt (Sigma W108), at a concentration of 16 μg/L, and the SERT inhibitor, fluoxetine HCl (Sigma W108), at concentrations of 3 and 4.5 μg/mL, both by immersion to males only. In these studies, the concentrations/doses and experimental designs we used were based on previous studies with these drugs in fish [37,38,47,86]. Further details of the pilot studies carried out in developing the methodologies used for the pharmacological manipulations are given in Additional File 5. There were no apparent effects of any of the compounds on the general level of behaviour/locomotion of the fish, which was consistent with the findings of other studies using these compounds in fish (e.g. [38,46]) and mammals (e.g. [85]).

### Data analysis

In the social interaction experiment, statistical differences in aggression between social ranks and sexes were tested by two-way ANOVA, followed by Holm-Sidak post hoc test, and between days by *t*-test (or a non-parametric alternative when appropriate) within Sigma Stat 3.10 (Jandel Scientific Software). Real-time PCR data were imported into GeneSpring GX 7.3 Software (Silicon Genetics, USA). All of the 40 study genes were expressed across the different regions of the brain in all fish, with two exceptions (*th2*, *gnrh2*) which we subsequently excluded from the analyses. Based on the entire set of samples for the total of 38 consistently expressed genes, genes and groups of samples were clustered following a hierarchical strategy with average linkage. The similarity measure used was Euclidian distance because this takes into account the magnitude of changes in gene expression. PCA was performed to identify the main trends between the gene expression profiles of individual fish. To identify differentially expressed genes, comparisons between social ranks, sexes and time periods were performed by *t*-test followed by multiple testing correction (Benjamini and Hochberg false discovery rate). In the pharmacological manipulations, the effect of each drug on aggression was tested by comparing the total number of aggressive acts between sham and experimental treatment for each fish via paired *t*-test (Sigma Stat 3.10). All differences were considered statistically significant at *P *< 0.05.

## Authors' contributions

AF and GP designed and carried out the social interaction experiment. AF carried out the gene expression analyses, data analyses and drafted the manuscript. GP and TH carried out the behavioural analyses. AF, TH and GP carried out the pharmacological manipulations. CT participated as a supervisor in the design of the studies and analyses, and edited the manuscript. All authors read and approved the final manuscript.

## Supplementary Material

Additional file 1 **Genes associated with aggressiveness in different regions of the brain**. Genes associated (*P *< 0.05) with aggressiveness in different regions of the brain in male zebrafish. Analyses were performed using data from dominant and subordinate males sampled on day 1 of aggression. Click here for file

Additional file 2 **Changes in the expression of individual genes in hypothalamus and telencephalon in males between day 1 and day 5 of the social interaction study**. Changes in the expression of individual genes in (A) hypothalamus and (B) telencephalon in males between day 1 and day 5 of the social interaction experiment. Data are represented as means ± SEM and expressed as the ratio of *'gene of interest'*:*rpL8*. Significant differences in expression are denoted by an asterisk (*P *< 0.05; *t*-test). Click here for file

Additional file 3 **Changes in the expression of individual genes in hypothalamus and telencephalon in females between day 1 and day 5 of the social interaction study**. Changes in the expression of individual genes in (A) hypothalamus and (B) telencephalon in females between day 1 and day 5 of the social interaction experiment. Data are represented as means ± SEM and expressed as the ratio of *'gene of interest'*:*rpL8*. Significant differences in expression are denoted by an asterisk (*P *< 0.05; *t*-test). Click here for file

Additional file 4 **Further details of the real-time PCR assays and methodologies used**. Word DOC displaying further details of the real-time PCR assays and methodologies used. Click here for file

Additional file 5 **Further details of the methodology used in the pharmacological manipulations**. Word DOC displaying further details of the methodology used in the pharmacological manipulations. Click here for file
