# Supplementary material for: Unravelling the neurophysiological basis of aggression in a fish model
Source: BMC Genomics. 2010 Sep 16;11:498. doi: 10.1186/1471-2164-11-498 (PMC2996994; doi:10.1186/1471-2164-11-498)

**Additional File 1.** Changes in the expression of individual genes in (A) hypothalamus and (B) telencephalon in males between day 1 and day 5 of the social interaction experiment. Data are represented as means ± SEM and expressed as the ratio of *‘gene of interest’*:*rpL8*. Significant differences in expression are denoted by an asterisk (*P*<0.05; *t*-test).


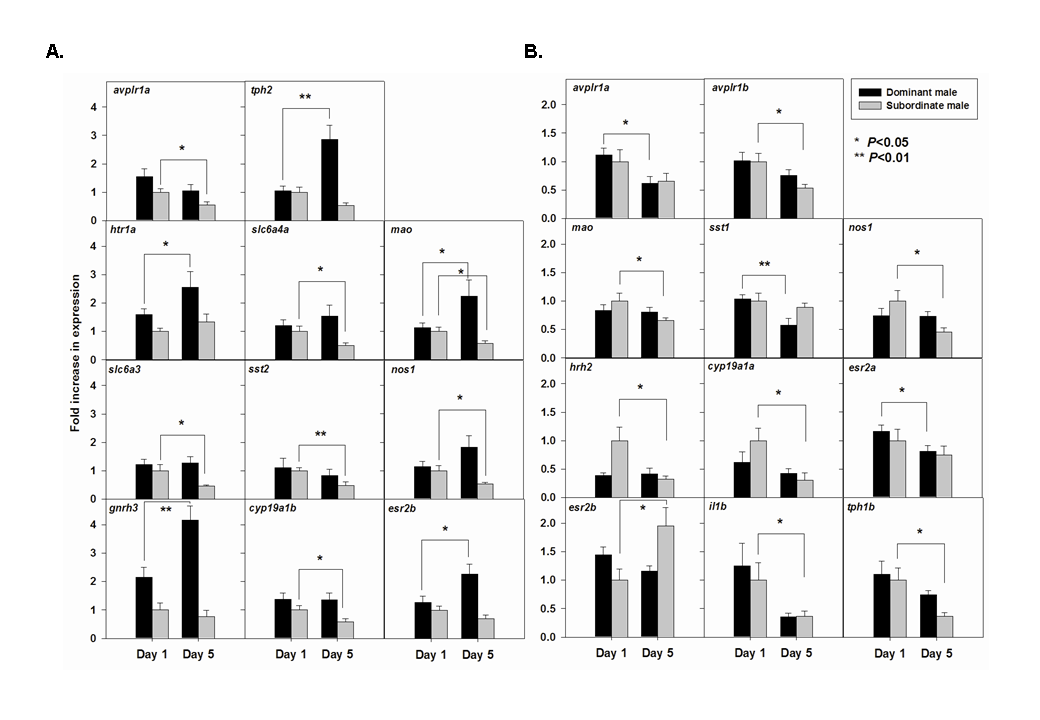

Supplement: Additional file 1 — Genes associated with aggressiveness in different regions of the brain. Genes associated (P < 0.05) with aggressiveness in different regions of the brain in male zebrafish. Analyses were performed using data from dominant and subordinate males sampled on day 1 of aggression. [file 1471-2164-11-498-S1.DOC]
