# Supplementary material for: Unravelling the neurophysiological basis of aggression in a fish model
Source: BMC Genomics. 2010 Sep 16;11:498. doi: 10.1186/1471-2164-11-498 (PMC2996994; doi:10.1186/1471-2164-11-498)

**Additional File 2.** Changes in the expression of individual genes in (A) hypothalamus and (B) telencephalon in females between day 1 and day 5 of the social interaction experiment. Data are represented as means ± SEM and expressed as the ratio of *‘gene of interest’*:*rpL8*. Significant differences in expression are denoted by an asterisk (*P*<0.05; *t*-test).


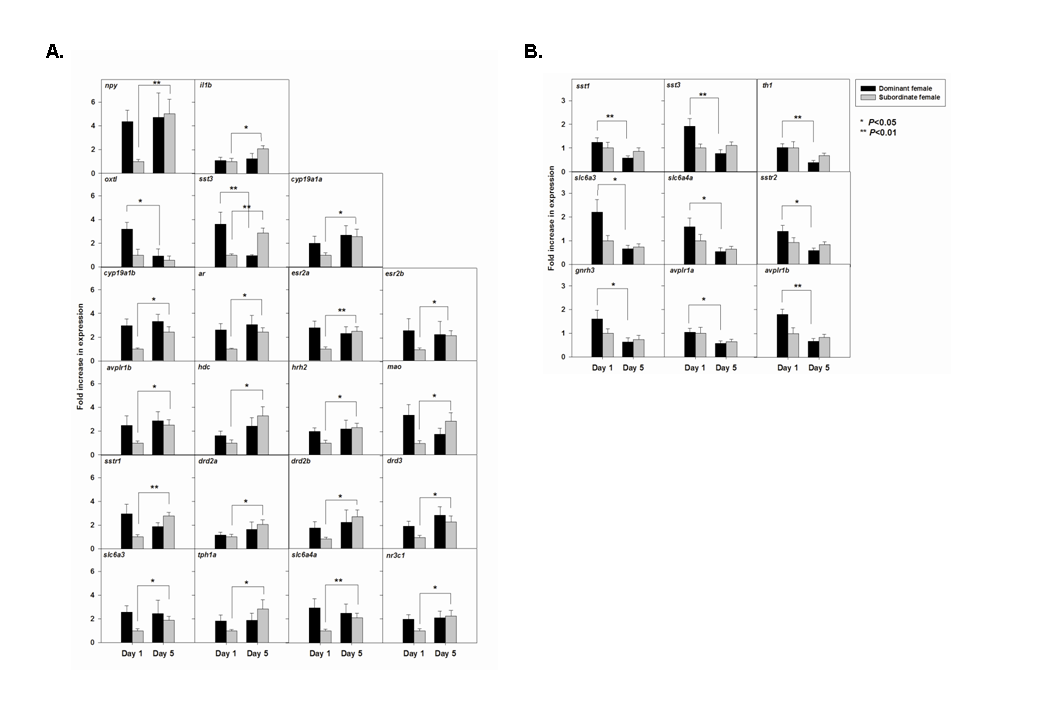

Supplement: Additional file 2 — Changes in the expression of individual genes in hypothalamus and telencephalon in males between day 1 and day 5 of the social interaction study. Changes in the expression of individual genes in (A) hypothalamus and (B) telencephalon in males between day 1 and day 5 of the social interaction experiment. Data are represented as means ± SEM and expressed as the ratio of 'gene of interest':rpL8. Significant differences in expression are denoted by an asterisk (P < 0.05; t-test). [file 1471-2164-11-498-S2.DOC]
