# Supplementary material for: Unravelling the neurophysiological basis of aggression in a fish model
Source: BMC Genomics. 2010 Sep 16;11:498. doi: 10.1186/1471-2164-11-498 (PMC2996994; doi:10.1186/1471-2164-11-498)
Supplement: Additional file 3 — Changes in the expression of individual genes in hypothalamus and telencephalon in females between day 1 and day 5 of the social interaction study. Changes in the expression of individual genes in (A) hypothalamus and (B) telencephalon in females between day 1 and day 5 of the social interaction experiment. Data are represented as means ± SEM and expressed as the ratio of 'gene of interest':rpL8. Significant differences in expression are denoted by an asterisk (P < 0.05; t-test). [file 1471-2164-11-498-S3.DOC]

**Additional File 3. Genes associated (*P*<0.05) with aggressiveness in different regions of the brain in male zebrafish. Analyses were performed using data from dominant and subordinate males sampled on day 1 of aggression.**

| **Region of brain** | **Gene** | **Direction of regulation in dominants** | **Fold-increase in dominants (mean±SEM)** | ***P*-value** |
| --- | --- | --- | --- | --- |
| Hypothalamus | *avpl* | Overexpressed | 4.31±1.60 | 0.038 |
|  | *avplr1b* | Overexpressed | 1.56±0.18 | 0.020 |
|  | *oxtl* | Overexpressed | 3.77±1.01 | 0.023 |
|  | *tph1b* | Overexpressed | 2.98±0.69 | 0.017 |
|  | *htr1a* | Overexpressed | 1.59±0.21 | 0.027 |
|  | *sst1* | Overexpressed | 1.56±0.21 | 0.048 |
|  | *sstr1* | Overexpressed | 1.67±0.22 | 0.019 |
|  | *th* | Overexpressed | 1.94±0.33 | 0.021 |
|  | *drd2c* | Overexpressed | 1.58±0.22 | 0.044 |
|  | *hdc* | Overexpressed | 1.88±0.17 | 0.003 |
|  | *hrh2* | Overexpressed | 1.73±0.28 | 0.046 |
|  | *crh* | Overexpressed | 1.75±0.26 | 0.033 |
|  | *nr3c1* | Overexpressed | 1.66±0.25 | 0.035 |
|  | *npy* | Overexpressed | 1.67±0.19 | 0.024 |
|  | *gnrh3* | Overexpressed | 2.16±0.34 | 0.015 |
|  | *esr2a* | Overexpressed | 1.52±0.18 | 0.030 |
|  | *ar* | Overexpressed | 1.96±0.30 | 0.010 |
| Olfactory bulbs/Telencephalon | *drd3* | Underexpressed | 0.56±0.09 | 0.041 |
|  | *slc6a3* | Overexpressed | 2.25±0.45 | 0.029 |
|  | *hrh2* | Underexpressed | 0.38±0.05 | 0.010 |
|  | *crh* | Underexpressed | 0.56±0.07 | 0.044 |
|  | *nr3c1* | Underexpressed | 0.62±0.05 | 0.023 |
|  | *npy* | Underexpressed | 0.68±0.08 | 0.029 |
|  | *gnrh3* | Overexpressed | 2.37±0.50 | 0.025 |
|  | *esr2b* | Overexpressed | 1.63±0.21 | 0.045 |
| Optic tectum | *tph1b* | Overexpressed | 1.87±0.26 | 0.009 |
|  | *htr1a* | Underexpressed | 0.60±0.11 | 0.048 |
|  | *sst3* | Overexpressed | 1.37±0.14 | 0.043 |
|  | *drd2b* | Underexpressed | 0.67±0.09 | 0.040 |
|  | *esr1* | Underexpressed | 0.67±0.10 | 0.032 |
| Hindbrain | *avpl* | Overexpressed | 2.765±0.807 | 0.021 |
|  | *tph1b* | Overexpressed | 2.367±0.551 | 0.043 |
|  | *sstr1* | Overexpressed | 1.395±0.127 | 0.031 |
|  | *slc6a3* | Overexpressed | 1.795±0.254 | 0.042 |
