# Supplementary material for: Unravelling the neurophysiological basis of aggression in a fish model
Source: BMC Genomics. 2010 Sep 16;11:498. doi: 10.1186/1471-2164-11-498 (PMC2996994; doi:10.1186/1471-2164-11-498)
Supplement: Additional file 4 — Further details of the real-time PCR assays and methodologies used. Word DOC displaying further details of the real-time PCR assays and methodologies used. [file 1471-2164-11-498-S4.DOC]

**Additional File 4. Details of the real-time PCR assays and methodologies used.**

The real-time PCR assays used in this study were optimised and validated for real-time PCR using SYBR Green chemistry as described previously [84]. Briefly, two sets of primers were designed for each target gene, to anneal at different positions within the transcript. Primer pair annealing temperatures were optimised for real-time PCR on a temperature-gradient program. Primer specificity was confirmed by gel electrophoresis, melt curve analysis and automated fluorescence sequencing of PCR products. To determine the detection range, linearity and real-time PCR amplification efficiency of each primer pair (i.e. validate the assay for quantitative purposes), real-time PCR amplifications were run in triplicate on a 10-fold serial dilution series of zebrafish brain cDNA and standard curves were calculated referring the threshold cycle (Ct; the PCR cycle at which fluorescence increased above background levels) to the logarithm of the cDNA dilution. Based on these results, the best-performing primer pair was selected for use in quantifying each of the target genes (see Table S1 for details).

Real-time PCR amplification of samples was performed in triplicate on each sample using 96-well optical plates (ABgene, Epsom, UK) with a 15 µL reaction volume containing 0.75 µL cDNA, 7.5 µL 2x ABsolute QPCR SYBR Green fluorescein mix (ABgene) , 6 µL molecular-grade water and 0.375 µL of each primer (MWG-Biotech; at a concentration of 10 pMol/µL). Hot start Taq polymerase was activated by an initial denaturation step at 95^o^C for 15 min followed by 40 cycles of denaturation at 95^o^C for 10 sec and annealing at the temperature determined separately for each primer pair during the optimisation process. Melt curve analysis was subsequently carried out for every amplification reaction and any reactions that did not generate the specific target product were excluded from the analyses. Reverse transcriptase-minus negative controls were run for a subset of the samples. Additionally, a template-minus negative control was run for each plate, and aliquots of zebrafish brain cDNA were repeatedly quantified on each plate to assess intra- and inter-assay variability.

To quantify differences in cDNA load between samples, target gene expression levels were normalised to a ‘housekeeping’ gene. In a preliminary study, the levels of a panel of ‘housekeeping’ genes (zebrafish *rpL8*, *ef1a*, *bactin*, 18S rRNA) were measured in a subset of samples using previously established methods [87]. *rpL8* exhibited the least variation between dominant and subordinate fish, and between day 1 and day 5 (and its expression did not significantly change between these conditions; *P*>0.05), and was therefore chosen for the normalisations. Relative expression levels (*target gene*: *rpL8*) were determined using a development of the arithmetic comparative method (2^-ΔΔCt^; [88]) that includes a correction for differences in PCR efficiency between the target and ‘housekeeping’ gene [89]. Results were initially expressed as relative expression ratios using the following formula:

*RE* = (*E ref*)^Ct^ *^ref^*/(*E target*)^Ct^ *^target^*

where *RE* is relative gene expression, *ref* is the ‘housekeeping’ gene, *target* is the gene of interest and *E* is PCR amplification efficiency for that particular gene.

Subsequently, to aid the visualisation of trends in the gene expression data (differences between dominant and subordinate fish), the data were divided by the mean of the ‘subordinate’ group, i.e. the ‘subordinate’ group was used as a calibrator sample and the relative gene expression value for each sample was expressed as the fold-increase from the calibrator group (set at a value of 1).

All real-time PCR assays for the quantification of the target genes has detection ranges of at least five orders of magnitude. Specificity of primer sets throughout this range of detection was confirmed by the observation of single amplification products of the expected size, melting temperature and sequence. All assays were quantitative with high efficiency values and correlation coefficients (see Table S4). Assays had a high level of precision and reproducibility with intaassay coefficient of variation (CV) of 2.42% (n=96). Where inter assay coefficient of variation was greater than average for a set of samples, plate-to-plate normalisations were carried out for the required target gene.

**Additional References**

**87.** Filby AL, Tyler CR: **Appropriate ‘housekeeping’ genes for use in expression profiling the effects of environmental estrogens in fish.** *BMC Mol Biol* 2007, 8:10.

**88.** Livak KJ, Schmittgen TD: **Analysis of relative gene expression data using real-time quantitative PCR and the 2^-ΔΔCt^ method**. *Methods* 2001, 25:402-408.

**89.** Soong R, Ruschoff J, Tabiti K: **Detection of colorectal micrometastasis by quantitative RT-PCR of cytokeratin 20 mRNA**. *Roche Diagnostics internal publication*, 2000. Lewes, UK: Roche Diagnostics Ltd.

**Table S1.** Real-time PCR primer sequences, product sizes, annealing temperatures (T_a_), efficiencies (*E*) and NCBI GenBank (http://www.ncbi.nlm.nih.gov/) accession numbers for the genes studied. Target gene names are given according to official zebrafish gene nomenclature guidelines (see http://www.zfin.org).

| **Target gene** | **GenBank accession no.** | **Sense primer (5’-3’)** | **Antisense primer (5’-3)** | **Product size (bp)** | **T_a_ (^o^C)** | ***E*** |
| --- | --- | --- | --- | --- | --- | --- |
| *avpl* | NM_178293 | CTGCTGCGATTCAGAGAG | CAGTTTTAGGCGATGTGTTC | 151 | 57.0 | 1.929 |
| *avplr1a* | XM_678600.2 | CAGCAGCGTCACCATCATC | GGAGCCCAGCACACAATG | 90 | 60.5 | 2.078 |
| *avplr1b* | XM_695195 | ACCGCTACATCGCCATC | GCTGCTGAGGACTAAACTG | 107 | 57.0 | 2.080 |
| *oxtl* | NM_178291 | GTGTGTGGACGGAGATG | GTGGGTGTGGCTTGAC | 97 | 57.0 | 1.956 |
| *tph1a* | AF548566 | CAGTTCAGTCAGGAGATTGG | GACAGTGCGTGCTTCAG | 176 | 57.0 | 1.934 |
| *tph1b* | NM_001001843 | TTATATTATTATCTGCCTTGTCTG | AGTGCTCTGTGGTATTTGG | 106 | 57.0 | 2.127 |
| *tph2* | NM_214795 | CAAGAGACAACAGCAACTATG | AAGCCCAACAGGTGATTTAG | 84 | 57.5 | 2.029 |
| *htr1a* | EH441641 | AGAGCAGCGAGGTGAC | GAGCCGATGATTTGGTAAC | 139 | 57.0 | 2.338 |
| *slc6a4a* | DQ285098 | AGTGGACCTGGGCAATG | AGAAGATACGGCAAGAGAAG | 81 | 57.5 | 1.641 |
| *mao* | NM_212827 | GCAGTCAGAGCCCGAATC | CACACCCATAAACTTGAGGAATC | 106 | 61.5 | 1.836 |
| *sst1* | AF435965 | GCCAAACTCCGCCAACTTC | GCTCCAGACGCACATCATC | 172 | 60.5 | 2.005 |
| *sst2* | NM_131727 | AGATACTAAAGAAGAGAGGAAGAC | AGGAAAAGGAAAGACTAAATGATG | 116 | 58.0 | 1.901 |
| *sst3* | BI473045 | GCTGTGATAATCGGTTGTAGAC | GGGCTGCTGGATGGAGAG | 123 | 59.5 | 1.822 |
| *sstr1* | XM_691574 | GCTACATCCTCATCATAGTCAAG | ACCACCATCATCACCATCAG | 106 | 59.0 | 2.075 |
| *sstr2* | NW_001510710 | TGTCGTGGTGGTGTTTGTG | AATGCGTAGAGGATGGGATTG | 162 | 60.5 | 2.040 |
| *sstr3* | XM_690273 | GCCTTGTGTCGCTCTTCTC | CTTCTTCCTCCTCGTCTTCTTC | 142 | 58.0 | 1.848 |
| *th* | AF075384 | AATCCACCATCTTGAAACC | TGCTAACATCCGACAGG | 101 | 54.5 | 2.026 |
| *th2* | NM_001001829 | AGCAAAACGGAGCAGTAAAGG | CTCATTAGAAAGGGCATACAACAG | 80 | 61.5 | 2.338 |
| *drd2a* | AY183456 | AGTGCCGTAAACCCAATC | GTATCATTTCCATCCCTTTCTG | 122 | 59.5 | 1.932 |
| *drd2b* | AY333791 | GTCTCCATCTCCGTCCTCTC | TTACCGAACACCACACAGAAG | 81 | 59.5 | 1.910 |
| *drd2c* | AY333792 | ATGCTCCTGACTCTCCTC | ATCTGCCACCGCCAAG | 126 | 57.0 | 1.988 |
| *drd3* | NM_183067 | TTCAGACCACCACCAACTACC | GCTCCGCCGACCACTTC | 106 | 61.5 | 1.643 |
| *slc6a3* | NM_131755 | ACTTTCTGCTGTCCGTCATC | GCTCCTCCGCCATTCTTG | 88 | 61.5 | 2.051 |
| *nos1* | NM_131660 | CGTATGGAAAGGAGTGAATGG | ATGAGTGTGGCGGAGAAC | 93 | 58.0 | 1.991 |
| *hdc* | EF150846 | TGCGAGGAAGGACCGAATC | CCACCAGGCGAGAGTTGAG | 86 | 61.0 | 1.949 |
| *hrh1* | NM_001042731 | TCGTCAGCCTCTCAATAGC | TCGCCACATAGTCCATCAC | 135 | 58.0 | 2.062 |
| *hrh2* | NM_001045338 | CACTGCTCCGCTACGATACC | TGAAACGCCCACCGAAACC | 91 | 61.0 | 2.251 |
| *crh* | NM_001007379 | CCACCGCCGTATGAATGTAG | GGGGACTGCCGCTCTC | 80 | 59.5 | 1.908 |
| *npy* | NM_131074 | TAAGACACTACATCAACCTCATAAC | GGCGGGACTCTGTTTCAC | 102 | 58.0 | 2.059 |
| *nr3c1* | EF567112 | GACTTGGTGGGTGGACTC | ACTGGTTGCTGATGATTTCTG | 103 | 58.5 | 2.104 |
| *il1b* | NM_212844 | GCTGGGGATGTGGACTTC | ACTCTGTGGATTGGGGTTTG | 98 | 57.0 | 2.303 |
| *gnrh2* | AF511531 | TTTACTCAACCGCCCACTTGAG | GCAGACCAGCACCATCACTTC | 140 | 64.0 | 1.960 |
| *gnrh3* | AJ304429 | TGTGTTGGAGGTCAGTCTTTG | CATTGGAGAATCAGCAGGAATAG | 142 | 60.5 | 2.140 |
| *kiss1* | EF641126 | CACCAAGAGGAGACAGAATG | GTAAATGATAAAACACCAAAGAAC | 151 | 57.0 | 1.820 |
| *ar* | NM_001083123 | ACGAGGGTGTTAGATGAGAC | AAGTATGAGGAAAGCGAGTAAAG | 129 | 58.0 | 1.968 |
| *esr1* | AB037185 | CGAGTGCCGCTGTATGAC | TGCTGCTGCTGGTTGTG | 130 | 59.5 | 2.040 |
| *esr2a* | NM_180966 | AGGAGAAAACCAAGTAAACCAATC | AGGCTGCTAACAAGGCTAATG | 173 | 59.0 | 1.856 |
| *esr2b* | AJ414566 | TGAGGAGATGGTGAACAAGAC | ACTGATGGATGGATGAATGAAATG | 131 | 57.8 | 2.180 |
| *cyp19a1a* | NM_131154 | AGCCGTCCAGCCTCAG | ATCCAAAAGCAGAAGCAGTAG | 101 | 61.5 | 2.119 |
| *cyp19a1b* | NM_131642 | TCGTTACTTCCAGCCATTCG | GCCTTCGTCATCACCATAGC | 75 | 59.5 | 1.944 |
| *rpL8* | NM_200713 | CCGAGACCAAGAAATCCAGAG | CCAGCAACAACACCAACAAC | 91 | 59.5 | 1.950 |
