# Supplementary material for: Unravelling the neurophysiological basis of aggression in a fish model
Source: BMC Genomics. 2010 Sep 16;11:498. doi: 10.1186/1471-2164-11-498 (PMC2996994; doi:10.1186/1471-2164-11-498)
Supplement: Additional file 5 — Further details of the methodology used in the pharmacological manipulations. Word DOC displaying further details of the methodology used in the pharmacological manipulations. [file 1471-2164-11-498-S5.DOC]

**Additional File 5. Further details of the methodology used in the pharmacological manipulations.**

In the development of the methodology for the pharmacological manipulations, we conducted several experiments to determine the most appropriate assay design for measuring effects on aggression, as follows:

**Effects of the presence of a female on male aggression**

To assess whether the presence of a female was necessary in the colony to promote aggression by the dominant male towards the subordinate male, 24 males were randomly assigned into 12 tanks, with 2 males per tank. To half of these tanks, one female was added. All colonies were left to acclimate overnight. The following two days, beginning at 10 am, each tank was observed for 6 min and the number of aggressive acts (a sum of chase, repel and bite) that the dominant male performed directed at the subordinate male was recorded. The level of aggression of the dominant male towards the subordinate male was compared between the tanks with females present and absent. There was no significant difference in the number of aggressive acts performed by the dominant male towards the subordinate male in tanks with a female present compared to tanks without a female (Fig. S1).


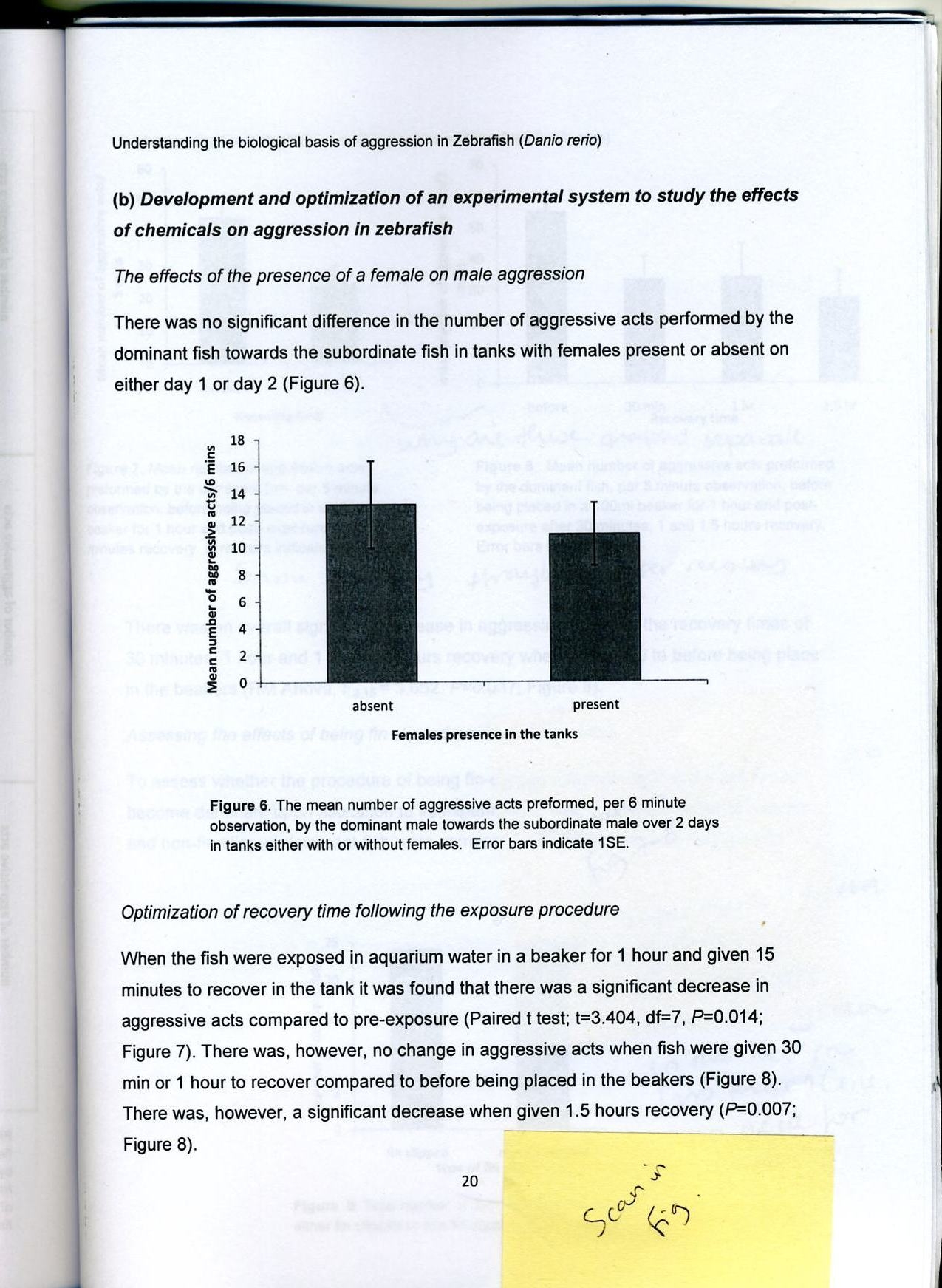


**Fig. S1. The number of aggressive acts performed per 6 min observation by the dominant male towards the subordinate male in tanks with and without a female. Data are presented as mean ± SEM.**

**Effects of the fin-clipping procedure**

To assess whether the procedure of being fin-clipped affected the subsequent aggression and/or social status of a fish upon allocation to its experimental tank, 24 males were randomly assigned into 12 tanks, with 2 males per tank. A small section of caudal fin was removed from one of the males in half of these tanks. All colonies were left to acclimate overnight. The following day, beginning at 10 am, each tank was observed for 6 min and the number of aggressive acts (a sum of chase, repel and bite) that the dominant male performed directed at the subordinate male was recorded together with the identity of each male (fin-clipped or non-fin-clipped). The level of aggression of the dominant male towards the subordinate male, and number of fish that were dominant compared to subordinate, was compared between the fin-clipped males and non-fin-clipped males. There was no significant difference in either parameter (data not shown).

**Determination of an optimal recovery time for the fish following the exposure procedure before assessing effects of the chosen compounds on aggression**

To determine an optimal recovery time for the fish following the exposure procedure, two experiments were conducted in which the treated fish were observed for 6 min and the number of aggressive acts (a sum of chase, repel and bite) that the dominant male performed directed at the subordinate male was recorded and compared to that after different recovery times. In experiment 1, a 15 min recovery time was trialled. In experiment 2, 30 min, 1 hr and 1.5 hr recovery times were trialled. In both experiments, the immersion route of administration (fish were placed individually into aquarium water in a glass beaker (50 mL volume) at 28 ^o^C for 1 h) was used.

In experiment 1, there was a significant decrease in the number of aggressive acts performed by the dominant male after a 15 min recovery time (Paired t-test; t = 3.404, df = 7, P = 0.014; Fig. S2). However, in experiment 2, there was no significant change in the number of aggressive acts performed by the dominant male after a 30 min or 1 hr recovery period (although a significant decrease in the level of aggression observed after 1.5 hr recovery; RM ANOVA; F3,15 = 3.652, P=0.037; Fig. S3). Based on these findings, and given that aggression gradually decreases after the spawning period which occurs immediately after the artificial dawn in zebrafish, we chose a 40 min recovery time for the experiments.


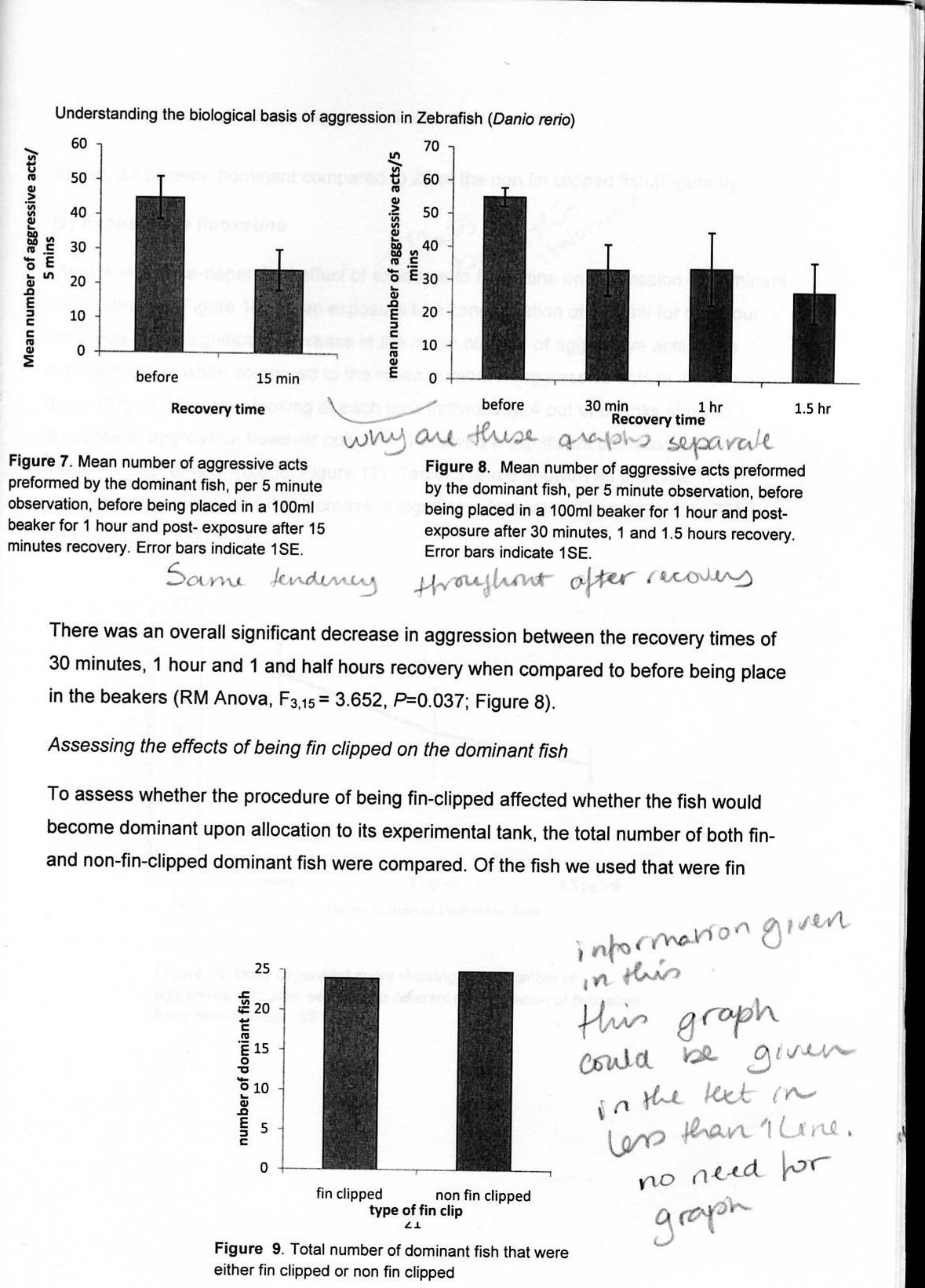


**Fig. S2. The number of aggressive acts performed per 6 min observation by the dominant male towards the subordinate male in tanks before and after the exposure procedure with a 15 min recovery time (experiment 1). Data are presented as mean ± SEM.**


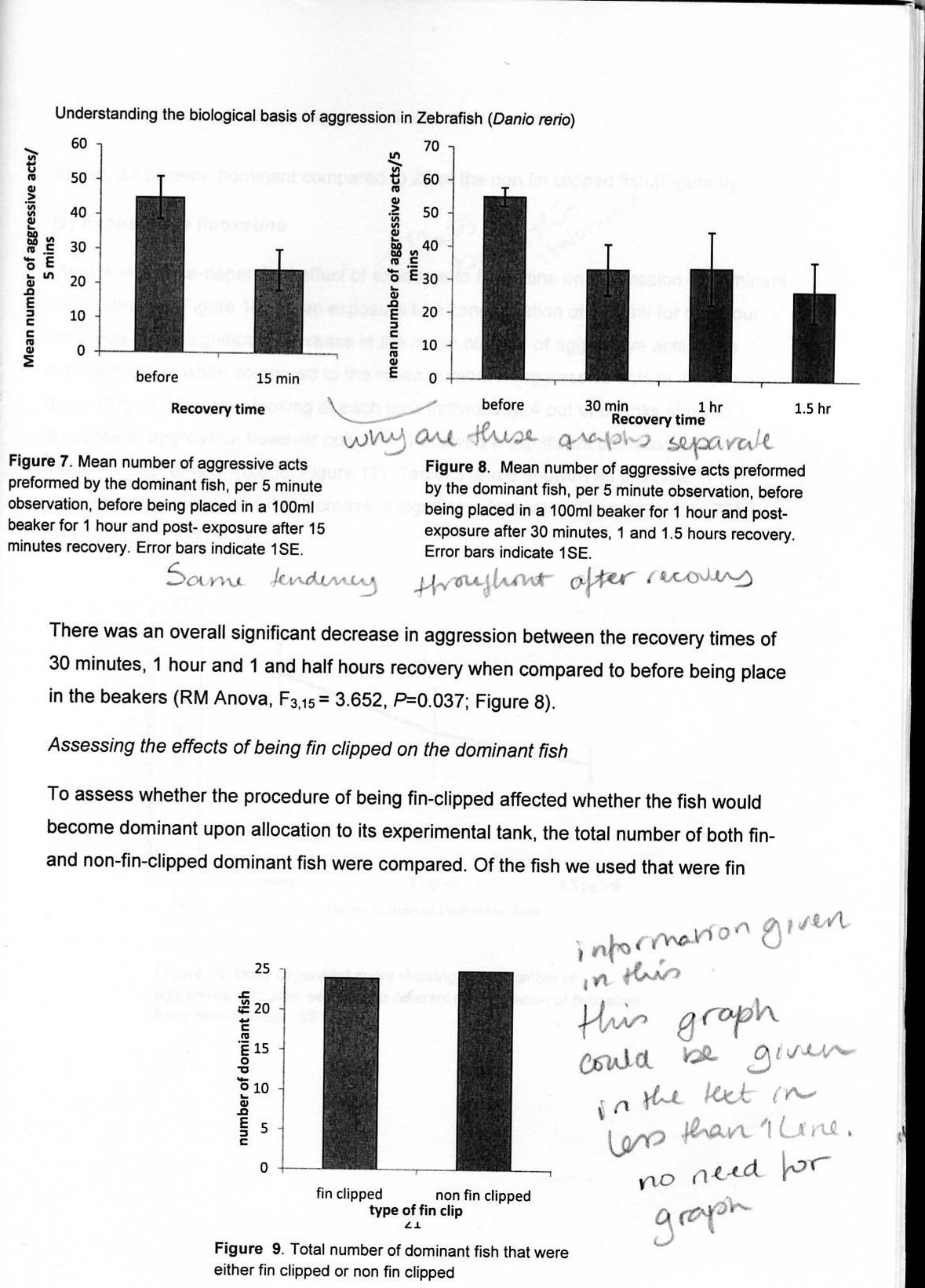


**Fig. S3. The number of aggressive acts performed per 6 min observation by the dominant male towards the subordinate male in tanks before and after the exposure procedure with 30 min, 1 hr, and 1.5 hr recovery times (experiment 2). Data are presented as mean ± SEM.**
